# Editorial: Women in animal nutrition and metabolism: 2021

**DOI:** 10.3389/fvets.2022.1090625

**Published:** 2022-12-20

**Authors:** Elsa Lamy, Asta Tvarijonaviciute, Lorena Franco-Martinez

**Affiliations:** ^1^Mediterranean Institute for Agriculture, Environment and Development, University of Évora, Évora, Portugal; ^2^CHANGE – Global Change and Sustainability Institute, University of Évora, Évora, Portugal; ^3^Institute of Research and Advanced Studies (IIFA), University of Évora, Évora, Portugal; ^4^Interdisciplinary Laboratory of Clinical Analysis (Interlab-UMU), Veterinary School, Regional Campus of International Excellence Mare Nostrum, University of Murcia, Campus de Espinardo, Murcia, Spain

**Keywords:** animal science, nutrition, women, research, metabolism

In science, as well as in other fields, gender equality is essential to ensure sustainable development as highlighted by UNESCO. Despite the growing number of women in science, globally we are still a minority, with <30% of researchers being women. If we focus on animal science, women make up only 40% of researchers (https://www.zippia.com/animal-scientist-jobs/demographics/) and, therefore, girls and women should be encouraged to pursue scientific careers.

The field of Animal Nutrition and Metabolism is broad and includes a number of important aspects, essential to understanding and promoting animal production and health. Moreover, and since animals may act as models, studies on animal nutrition and metabolism are also of major relevance for advances in human physiology and health.

The field of Animal Nutrition and Metabolism includes very diverse topics that go from ingestion and feeding choices to biological bases of animal nutrition and metabolism, including the effect of foods and food compounds in physiology, as well as metabolic disorders resulting from food intake or nutrient imbalance. In the last years, with the advance of different analytical techniques, the knowledge about nutrition and metabolism increased. Moreover, the use of different types of samples from invasive and non-invasive origins allowed the clarification of different nutritional and metabolic processes. Another important point that has emerged in recent years is animal wellbeing and the guarantee that animal production is sustainable and ecological. Approaches to access animal stress and change environmental conditions, to assure wellbeing, are being promoted. As well, due to the alarm coming from climate change observations, the focus on ruminant digestion as a source of greenhouse gases increased research about nutritional approaches aimed at minimizing the effect of these species. Food products and additives able to promote production while reducing methane emissions are being tested in different laboratories.

Along the same line, in pets, aspects like nutrition and the maintenance of adequate metabolism are of utmost relevance. In line with humans, and sometimes as a response to them, pets are facing an increase in metabolic problems, some of them greatly associated with nutritional aspects. For example, obesity in dogs and cats has increased considerably in the last years, and several studies highlight this having a close relationship with owners' food and physical activity habits. This rise in obesity has the consequent result of a rise in metabolic problems, like glucose intolerance and other associated obesity-related metabolic pathologies, with an increase in morbidity and mortality of these animals.

This Research Topic presents a collection of manuscripts authored by women, focusing on different aspects of animal nutrition and metabolism.

The study from Cavanaugh et al. is the only one, in this Research Topic, performed on dogs. This study presents results about the consumption of a plant-based diet for dogs, in the levels of circulating trimethylamine N-oxide (TMAO), which is a molecule derived from gut microbiota. The focus on TMAO was done following the previous observation that elevations in its circulating levels, or in the levels of its precursors, are associated with heart failure. In this study, 16 healthy adult mixed breed dogs took part in a randomized, 2-treatment, 2-period crossover weight-maintenance study, where animals were fed on the plant-based diet or the conventional diet (containing components of vegetable and animal origin) for 4 weeks. After measuring the plasmatic levels of TMAO and of two of its precursors (choline and betaine), it was concluded that, although not reducing TMAO plasmatic levels, the change to a plant-based diet resulted in the decreased plasmatic levels of choline and betaine, in healthy dogs. Although more studies are needed to see the potential of these diets on diseased animals, the results suggest potential health benefits to the consumption of these diets are also interesting from an environmental sustainability point of view.

The remaining four original studies included in this Research Topic are focused on ruminants: two of them consider different aspects of goats' nutrition and the other two focus on bovines, either for milk or for meat production.

The prevalence of mycotoxins in feed is one of the major concerns of producers, particularly in humid and rainy areas. Mycotoxins can affect animal health, at different levels, and can also contaminate animal products, being also a concern for human health. Because of that, Wu et al. evaluated the effect of supplementing a mycotoxin-contaminated diet with functional oligosaccharides (particularly galactomannan and mannan oligosaccharides) on the growth and health of Xiangdong goats. This study presented promising results since the authors were able to observe that the supplementation with galactomannan oligosaccharides resulted in goats' protection in different ruminal fermentation parameters, by promoting the proliferation of beneficial rumen bacteria. Moreover, this supplementation also resulted in the alleviation of mycotoxin injury of the liver and kidney, suggesting that this may be a cost-effective strategy for livestock in regions where mycotoxins are a problem.

Also working with the nutrition of goats, Mi et al. tested the effect of low-protein diets on the levels of calcium in the blood and their correlation with bone metabolism. This study was motivated by the fact that low-protein diets are commonly used in goat nutrition due to the high pressure on producers to reduce costs and nitrogen emissions. However, dietary protein is essential for animal growth, and besides ruminants being greatly dependent on microbial protein, they still need proteins from feed, which are also a vital source of synthetic osteoprotein amino acids. By allocating 24 Xiongdong black goats to two treatments (control diet vs. low protein-diet), for 36 days, the authors observed no changes in blood calcium, nor in bone metabolism markers, including the markers reflecting the bone absorption and formation. According to the authors, compensatory homeostasis mechanisms can occur, in goats, to maintain a stable bone metabolism, presenting also a more detailed hypothesis for explaining these results. As such, concerning the specific case of bone metabolism, reducing the levels of dietary protein in goats may not be a major problem.

Dietary interventions in dairy cattle are greatly studied, since diet is critical, in these animals, to allow them to achieve all their productive potential and wellbeing. Because of that, Liu et al. tested the effect of supplementation with N-carbamoylglutamate on the digestibility, rumen fermentation, milk quality, antioxidant parameters, and metabolites of Jersey cattle when present in high-altitude areas of Tibet. The Jersey cattle, is a breed with high milk production, being immediately after Holstein in productive potential, that has the major advantage of being highly resistant to disease, being able to maintain production even on rough feed, having a high feed utilization rate, and high milk fat content. For all of this, this breed is chosen for production in areas like Tibet. Despite that, the high altitude of Tibet (average altitude of 4,000 m) makes animals in this region to be prone to different pathophysiological changes caused by high-altitude hypoxia. Dietary solutions, such as the use of additives, to minimize this problem and guarantee animal health, wellbeing and productivity are of major importance. N-carbamoylglutamate, able to promote the synthesis of endogenous arginine, which, in turn, is known for its beneficial effects on immune function, was applied as a feed additive to Jersey cattle, in the referred study. The authors observed that an amount of 20 g/day, per animal, has a positive impact, regulating the levels of thyroxine, transferrin, and endothelin and improving the blood oxygen saturation of these animals, at high altitudes. These positive effects, without affecting nutrient digestion and immunity are promising for dairy animal nutrition.

Also, in the area of bovine nutrition, Brito et al. present interesting results about how the composition of cattle feed can influence the sensory quality of meat products. In this precise case, the authors assessed the effect of diets containing different oilseed grains on the fatty acid profile and sensory characteristics of a burger, at 0, 30, 60, 90, and 120 days of storage. It was possible to conclude that sunflower and soybean grains are able to provide positive results in terms of hypocholesterolemic/hypercholesterolemic ratio without negatively influencing the concentrations of n-3-fatty acids. In these cases, the maximum storage period that can guarantee the sensory quality of this product is 30 days. These results and this type of study are of particular importance in the current context, where healthy, nutritional, and sustainable diets are warranted and where animal nutrition may have a particularly important role to achieve them.

Overall, the studies presented in this Research Topic, led by women, focus on different aspects of Animal Nutrition and Metabolism, showing the relevance of this area in different aspects of production, health, and wellbeing for both animals and humans.

## Author contributions

All authors listed have made a substantial, direct, and intellectual contribution to the work and approved it for publication.

